# Imidacloprid Uptake and Accumulation in Lettuce Plant (*Lactuca sativa* L. var. *longipolia*) and Its Effects on Abundance of Microbial Communities in Cultivated and Non-Cultivated Arid Soil

**DOI:** 10.3390/plants13152017

**Published:** 2024-07-23

**Authors:** Ahmed A. Ahmed, Abdulgader Bazyad, Fahad Alotaibi, Khaled D. Alotaibi, Garry Codling, Hattan A. Alharbi

**Affiliations:** 1 Department of Plant Protection, College of Food and Agriculture Sciences, King Saud University, P.O. Box 2460, Riyadh 11451, Saudi Arabia; aahmed5@ksu.edu.sa (A.A.A.); abazeyad@ksu.edu.sa (A.B.); 2 Department of Soil Science, College of Food and Agriculture Sciences, King Saud University, P.O. Box 2460, Riyadh 11451, Saudi Arabia; fanalotaibi@ksu.edu.sa (F.A.); khalotaibi@ksu.edu.sa (K.D.A.); 3 Centre for Resilience in Environment, Water and Waste (CREWW), University of Exeter, N. Park Road Exeter, Devon EX4 4QE, UK; g.codling@exeter.ac.uk

**Keywords:** imidacloprid, neonicotinoids, lettuce, uptake, translocation, degradation, microbial activity

## Abstract

Systemic plant protection products, such as neonicotinoids (NIs), are capable of being translocated throughout a plant. Although NIs are less toxic to mammals, fish, and birds, their impact on microbial and non-target insects is of concern. This study investigates the uptake, translocation, and accumulation of the NI, imidacloprid (IMI), in romaine lettuce (*Lactuca sativa* L. var. *longipolia*). Exposing 15-day-old seedlings to “10 mg/L” of IMI, the effects on microbial communities in both cultivated (CS) and non-cultivated soil (NCS) were studied along with IMI translocation within plant tissues. The concentrations of IMI in soil varied temporally and between soil types after initial application, with a decrease from 2.0 and 7.7 mg/kg on the first day of sampling to 0.5 and 2.6 mg/kg on the final sampling day (day 35) for CS and NCS, respectively. The half-life of IMI soil was 10.7 and 72.5 days in CS and NCS, respectively, indicating that IMI degraded more quickly in CS, possibly due to smaller grain size, aeration, microbial degradation, and water flow. The accumulated concentrations of IMI in lettuce tissues ranged from 12.4 ± 0.2 and 18.7± 0.9 mg/kg in CS and NCS, respectively. The highest concentration of IMI was found in the shoots, followed by the roots, whereas the soil showed the lowest IMI residuals at the end of the trial. Soil bacteria and fungi were altered by the application of IMI, with a lower abundance index within the bacterial community, indicating a negative impact on the distribution of bacteria in the soil.

## 1. Introduction

Neonicotinoids (NIs) represent a class of systemic insecticides structurally analogous to nicotine. Their widespread utilization has raised significant concerns regarding their impact on non-target organisms, notably the honeybee (*Apis mellifera*). In the United Kingdom, approximately 30% of cultivated land employs some form of NI, and globally, NIs represent a quarter of all plant protection products. It is estimated that the NI marketplace is worth nearly 4 billion USD annually [1]. Used globally, particularly in seed treatments across various crops, NIs have gained favor owing to their lower toxicity to vertebrates relative to organochlorine or organophosphate insecticides, as well as their systemic mode of action and ease of application [2,3,4]. The systemic nature of NIs facilitates their transport throughout the entire plant following application via seeds, aerial and ground sprays, chemigations, or injections, ensuring that any part susceptible to insect feeding contains detectable levels of NIs [5]. Upon ingestion by insects, NIs bind irreversibly to acetylcholine receptors (nAChRs), inducing paralysis and eventual mortality. Although all animals possess nAChR receptors, they are less dominant and exhibit weaker interactions with the nervous system in vertebrates. It is estimated that NIs are up to ten times more potent toward insects compared to other animals [6]. However, some NIs, such as imidacloprid (IMI), with a maximum residual limit (MRL) in lettuce of 2 mg/kg can persist in the soil for extended periods, leading to potential long-term environmental exposure [2,7]. The high aqueous solubility of NIs can also facilitate their transport into aquatic ecosystems, leading to farm ponds containing significant concentrations of NIs, and the potential for long-range aquatic transport from agricultural runoff [8]. NI residues in soil or water can also be absorbed by non-target vascular plants, increasing the potential for human dietary exposure and affecting unintended organisms [9,10,11,12]. The potential for unintended impacts on non-target organisms and the environment raises concerns regarding the of global mass NIs used [13,14]. uptake of NIs, like IMI, by plants and translocation within their system, can differ markedly between plant species with tissue specific concentrations observed [4]. Investigations into IMI uptake across different plant species, including sunflowers, wheat, and cotton, have revealed differences in how these pesticides are absorbed and translocated [15,16]. Research on clothianidin (CLO), another NI in maize, focused on the translocation of NIs from soil to root, shoot, and seed and observed variability between tissues, with only a 2% retention rate; this raises many questions regarding the efficacy of application and environmental exposure [17]. The transport dynamics of NIs through plant root systems and uneven distribution between tissues can be influenced by various factors, including soil properties and agricultural management practices.

Continuous cultivation and management practices influence several soil properties, including organic matter content, pH, cation exchange capacity, nutrient status, microbial size and activity, bulk density, water holding capacity, and soil structure [18,19,20]. Intensive farming based on synthetic pesticides and mineral fertilizers has led to the loss of soil biodiversity, which contributes to the suppression of plant pathogens [21]. Microbial activity is often used as an early and sensitive indicator of changes in soil quality [22,23]. Soil microbial biomass, activity, and species are also influential in the rate and type of degradation of agrochemicals. Pesticide use impacts the soil microbiome and may restrict the functions and role of microbes in agroecosystems. Previous studies have indicated that IMI and its metabolites have limited effect on altering the soil microbiome [24,25].

Research investigating the uptake or translocation of imidacloprid in romaine lettuce from soil with long-term cultivation compared to non-cultivated soil are limited, and largely focus on Europe or the Americas. The arid soils of Saudia Arabia (SA) are typically sandy, low in organic matter, and characterized by minimal fertility and moisture retention and have been less well studied. However, arid soil that has been cultivated for long periods are expected to have a higher content of organic matter and nutrients, compared to uncultivated soil, and this can stimulate microbial activity and affect pesticide breakdown in soil and, therefore, their uptake by plants. Such information is necessary to determine how cultivated (CS) and non-cultivated (NCS) soils differ in their effects on pesticide breakdown and transport into plant tissues, especially in arid climates. In addition, SA’s farming productivity has increased massively in the last decade leading to greater reliance on NIs and fertilizers. Therefore, the aim of this study was to investigate the uptake and accumulation of IMI within lettuce plant tissue and IMI distribution in cultivated (CS) and non-cultivated (NCS) arid soils, in addition the impact of IMI on the composition and dynamics of soil microbial communities in both soil types is also investigated. The findings of this study will improve our understanding of the accumulation and distribution of neonicotinoids in vegetables grown in arid soils, varying in their basic characteristics as a function of the cultivation period. Understanding the breakdown and distribution of these pesticides in these soils will contribute to food safety and pesticide management under the conditions of these soils.

## 2. Materials and Methods

### 2.1. Chemicals and Reagents

Imidacloprid Standard (IMI) 98.6% was obtained from Sigma-Aldrich Merck KGaA (Darmstadt, Germany). Imidacloprid 17.8 SL formulation (Bayer Crop Science Ltd., Barmen, German), was purchased from Alzanan Agro-chemical Co. Ltd, (Al-Riyadh, KSA). Acetonitrile (HPLC Grade) was purchased from Agilent Technology (Santa Clara, CA, USA). QuECHERS (Quick, Easy, Cheap, Effective, Rugged, and Safe) extraction kits were used, containing anhydrous magnesium sulfate (MgSO_4_), sodium chloride (NaCl), and primary secondary amine (PSA) were purchased from United Chemical Technologies, Inc. (Bristol, PA, USA). Trypticase soy agar (TSA) and potato dextrose agar (PDA), were obtained from Sigma-Aldrich Merck KGaA (Darmstadt, Germany).

Lettuce seeds “Romaine lettuce seeds” were obtained from AL-TUWAIJRI-AGRI & TRADING. Co., Ltd. (Al-Riyadh, Saudi Arabia) and were not pretreated with IMI or other plant protection products.

### 2.2. Soil Collection and Preparation

The soils used in the current study were collected from Huraymila area near Riyadh, Saudi Arabia. The cultivated soil (CS) was collected from a private farm that has been under cultivation for more than 10 years. The virgin soil, referred to here as non-cultivated soil (NCS) was collected from a land in the vicinity of the cultivated soil. Both CS and NCS samples were collected from a depth of 30 cm. Soil samples were collected in large containers, allowing for a sufficient quantity of soil to be obtained for planting while ensuring that the samples were representative of the respective areas from which they were taken. The collected soil samples were air-dried, sieved through a 2 mm mesh screen, homogenized manually, and stored in the greenhouse.

#### Soil Analysis

Soil texture was determined using hydrometer method where particle size was distributed to determine the percentage of silt, clay, and sand in the soil samples [26]. The organic matter content was determined using the method described by Nelson and Sommers (1996) [27]. Nitrogen, phosphorus, and potassium were measured according to the methods as detailed in previous studies [27,28]. The pH and EC were determined in a 1:2.5 soil: distilled water suspension using pH and EC meters [27]. 

### 2.3. Planting, Growth Conditions, and Harvesting

The lettuce seeds were germinated for 3 days at 30 °C on filter paper in a lab, and then 5–7 seedlings were transferred to pots with dimensions (18 cm length, 15 cm width, 12 cm depth) filled with 0.8 kg of soil sample. In total, 84 pots with plants and 42 control pots without plants were prepared. After 15 days of growth, the lettuce was thinned to retain three similar-sized plants in each pot. Seedlings were divided into CS and NCS groups and further divided into control (water with no IMI) and exposed seedlings. The pots were divided into two groups. The first two groups of soil were separately mixed with 120 mL of 10 mg/L of IMI [29]. The third group served as the control and received only 120 mL of distilled water to ensure the same moisture level across all experimental pots. All pots were kept in a greenhouse for 35 days. Throughout the 35 days in the greenhouse, they were irrigated with 100 mL of water every four days.

At seven periods after exposure (1, 5, 10, 15, 21, 28, and 35 days), triplicate pots were harvested at random from each group, and care was taken to harvest at approximately the same time of collection. The roots and shoots of the seedlings from each sample pot were separated and cut into ~1 mm pieces, and both soil and plant samples were stored in separate amber glass vials at −20 °C until analysis.

### 2.4. Extraction, Purification, and Analysis

Lettuce extraction and clean-up were based on the QuECHERS sample preparation method for pesticides [13]. In brief, lettuce tissues (roots or shoots) were equilibrated to ambient temperature, homogenized with a solvent-cleaned blender, and 3 g was subsequently placed in a 15 mL Teflon centrifuge tube. Ten mL of acetonitrile was added, followed by a 5 min incubation period, before being manually agitated for one minute, vortexed for one minute, and centrifuged for 5 min at 2655.25 × 10^−5^ g. For purification, 1 mL of the supernatant was transferred to a 2 mL centrifuge tube containing 50 mg PSA and 150 mg MgSO_4_. The mixture was vortexed for 1 min and centrifuged under the same conditions as previously described. Finally, 1 mL of the purified extract was filtered through a 0.22 mm membrane into a sample vial for analysis.

For soil samples, 5 g, were extracted with 10 mL of acetonitrile initially left for a 5 min incubation period. The sample was subsequently vortexed for 1 min and centrifuged for 5 min at 2655.25 × 10^−5^ g. Then, 2 mL of supernatant was combined with 2 g NaCl, agitated, and centrifuged. The resulting extract was filtered through a 0.22 mm membrane into a sample vial for analysis.

### 2.5. Chromatographic Conditions

The detection and quantification of IMI was carried out using Waters 2545 HPLC Binary Gradient Module (BGM) equipped. with a Photodiode Array Detection (PDA) 2489 UV/Vis Detector (Framingham, Massachusetts, NE, USA). Chromatographic separation was carried out using a MEDITERNEAN C18 column with dimensions of 250 × 4.6 mm and a pore size of 5 μm (Element, London, UK). The mobile phase consisted of HPLC-grade acetonitrile (A) and water (B). The flow rate was maintained at 1.2 mL/min, with a 20 μL injection volume, and the column oven was maintained at 30 °C. Photometric detection was performed at 270 nm to quantify IMI. The retention time of IMI was observed at 3 min, and the UV-Vis spectra, along with the retention time, were utilized for compound identification.

### 2.6. Soil Microbial Characterization

Total heterotrophic bacteria and fungi were enumerated using the dilution plate count method [30]. Briefly, 10 g of dry soil was immersed in 90 mL of sterile Phosphate-Buffered Saline (PBS) solution. The sample was placed in a bench shaker for 30 min at 175 rpm, and subsequently, ten-fold serial dilutions were performed. A 1 mL aliquot of the dilutions 10^−3^, 10^−4^, 10^−5,^ and 10^−6^ for bacteria and 10^−2^, 10^−3^, and 10^−4^ for fungi were transferred into sterilized petri dishes followed by pouring media. The pouring media consisted of autoclaved 1/10-strength trypticase soy agar (TSA) and potato dextrose agar (PDA) for the enumeration of bacteria and fungi, respectively. Plates were incubated at 28 °C; bacteria were counted after 24, 48, and 72 h, while fungi were counted after 48, 72, 96, and 120 h. Counts were carried out in triplicate and expressed as the number of colony-forming units per g of dry soil (CFU g^−1^ soil).

### 2.7. Quality Assurance and Quality Control

As detection of IMI was performed via a UV/Vis detector, the addition of mass-labeled IMI for extraction recovery was not feasible; therefore, fortified recoveries were performed using ‘clean’ soil and lettuce samples.

The working standard (stock solution) of IMI (100 mg/L) was prepared in acetonitrile in the range of 0.1–5.0 mg/kg for HPLC-PAD analysis, and a working solution was prepared fresh through serial dilution of stock ranging from 0.01–3.00 mg/kg for HPLC-PDA analysis, and the matrix-matched to CS and NCS. (r2 of calibration curves ≥ 0.99 for acceptability).

The precision of the system was observed to assess the repeatability, which is expressed as a percentage of the relative standard deviation (%RSD). %RSD was calculated from six replicates of the pesticide.

%RSD = (Standard deviation/Mean peak area) × 100

At the lower concentration (0.1 mg/kg), the RSD value was 5.7% to 6.0% in both CS and NCS, respectively. At the higher concentration (0.5 mg/kg), the RSD value is 13.2% to 3.1% in CS and NCS, respectively. The accuracy is expressed as % recovery, which was calculated using the following equation:

%Recovery = (Detected concentration/Nominal concentration) × 100

At the lower concentration (0.1 mg/kg), the percentage recovery ranged from 106% to 99% in both CS and NCS, respectively. At the higher concentration (0.5 mg/kg), the recovery ranged from 85% to 86% in CS and NCS, respectively. The limit of detection (LOD) and limit of quantification (LOQ) of the IMI were calculated as follows: LOD was 0.12 and 0.05 mg/L for CS and NCS, respectively, and LOQ was 0.36 and 0.17 mg/L for CS and NCS, respectively.

### 2.8. Concentration Factors for Uptake

#### 2.8.1. Root and Translocation Factors

The root concentration factor (RCF) and translocation factor (TF) are generated by the ratio of IMI in the root to that in soil for RCF [16]. and concentration in the shoot to that in the root for TF [31,32]. RCF factors over one indicate a propensity for compounds to move into the plant roots over soil, and for a TF, a value greater than one indicates preferential transport to the shoots.

#### 2.8.2. Temporal Shift in IMI in Soil

The concentration of the compound in the soil over time (*Ct*), is calculated as follows:

*Ct* = *C*_0_ × *e*^−*kt*^

where:

*Ct* = concentration of the compound at time *t* (in mg/kg), *C*_0_ = initial concentration of the compound (in mg/kg), *k* = first-order rate constant (in 1/day), and *t* = time in days. This formula estimates how the concentration of a compound changes in the soil over time. Additionally, the dissipation half-life t12 is calculated using the first-order rate constant (*k*) according to [13]. as follows:

t12=ln 2k=0.693k

where:

t12 = dissipation half-life, (*ln*_2_) = natural logarithm, and (*k*) = first-order rate constant.

The dissipation half-life indicates the time it takes for the concentration of the compound in the soil to reduce by half.

### 2.9. Statistical Analysis

To compare the differences in the uptake and translocation of IMI during different periods of 35 days and soil “types”, an independent *t*-test with *p* < 0.05 was applied using GraphPad Prism software (version 8).

## 3. Results

### 3.1. Soil Physical and Chemical Parameters

The basic characteristics of the two soils (CS and NCS) are provided in Table 1. Both soils were dominated by a sandy particle size; however, in NCS, only 10% was silt compared to 30% in CS, with a larger proportion of sand. The CS showed higher content of organic matter (1.79 %) than that observed in NCS (0.55 %) (Table 1). A significant higher content of total nitrogen and available phosphorus was also found in the CS. 

### 3.2. Imidacloprid (IMI) Concentration in Soil

**Figure 1:**
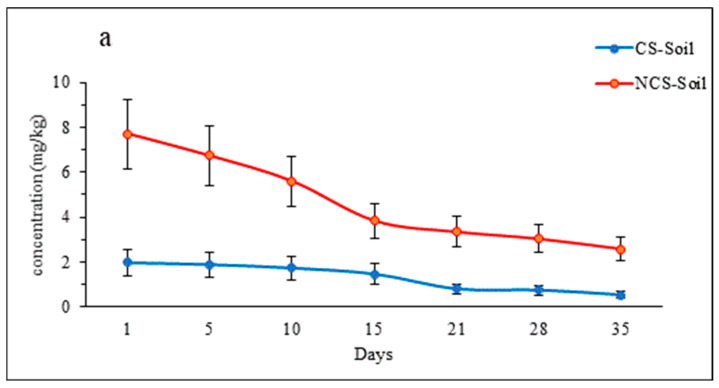

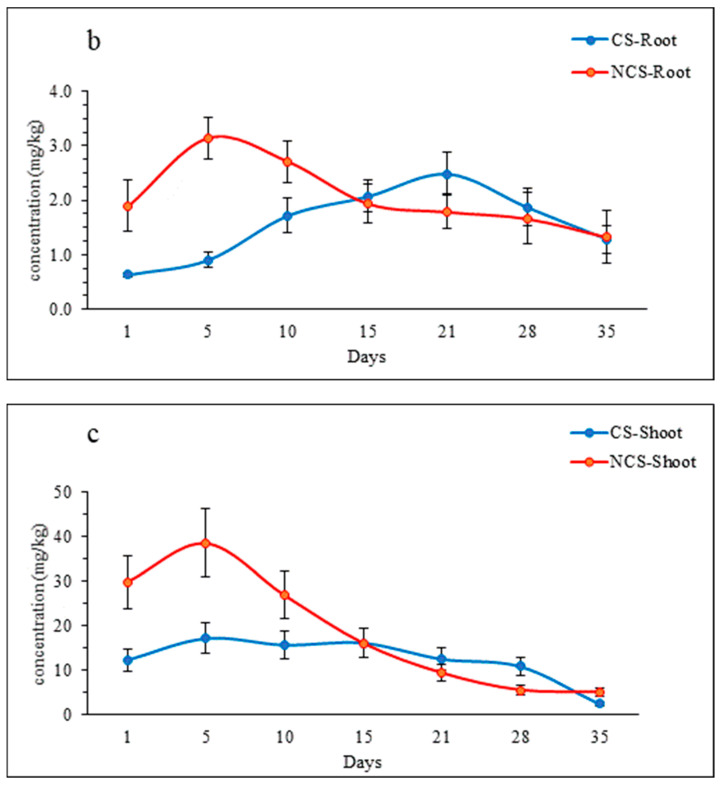
The concentration of IMI in the soil (**a**), root (**b**), and lettuce leaf (**c**), and where: (CS) = cultivated soil, (NCS) = non-cultivated soil. Each point is the mean of three replications ± standard error.

The concentration of IMI in the soil varied temporally and among the soil types, with NCS showing the highest concentration of IMI after 1 day of application (Figure 1a). The concentration of IMI was 7.7 and 2.0 mg/kg on day 1 and decreased to 2.6 and 0.5 mg/kg on day 35 for NCS and CS, respectively. Overall, this represents a 52.7% and 50.8% reduction in the IMI concentration over the study period for CS and NCS, respectively.

### 3.3. IMI Uptake by Lettuce

The uptake rate of IMI in NCS lettuce may reflect more abundant IMI in the soil at the beginning of the experiment. The average concentration of IMI in lettuce roots was 1.6 mg/kg in CS and 2.1 mg/kg in NCS, indicating that a similar level is achieved irrespective of external abundance, although the rate of achieving equilibrium differs.

Once IMI is taken up by the plant roots, it is transported from the roots to the shoots through translocation [16]. A rapid increase in concentration was observed during the first few days of exposure (Figure 1c). In both soils, the highest concentration of IMI in shoots was reached after approximately 5 days of exposure. The average concentrations of IMI in plant shoots were reported as 12.4 mg/kg in CS and 18.7 mg/kg in NCS. This varies from the roots, where the maximum concentration in CS was reached at approximately 21 days.

During the cultivation period (excluding day 1), the IMI concentration in lettuce shoots was greater than that in the corresponding soil and plant roots.

### 3.4. Root Concentration Factor (RCF) and Translocation Factor (TF)

The root concentration factor (RCF) is used to evaluate the ability of a plant to take up a pesticide from the soil to the root system [31,33,34]. The RCF values for IMI over the study period ranged from 0.3 to 2.5 for CS and 0.3 to 0.5 for NCS (Table 2). The average RCFs of IMI in CS and NCS were calculated as 1.6 and 0.5, respectively. The TF values for IMI were significantly smaller on the first day of exposure compared to the subsequent days. The average TFs of IMI in CS and NCS were calculated as 9.7 and 8.3, respectively.

### 3.5. Effects of Imidacloprid on Soil Microbial Communities

**Figure 2:**
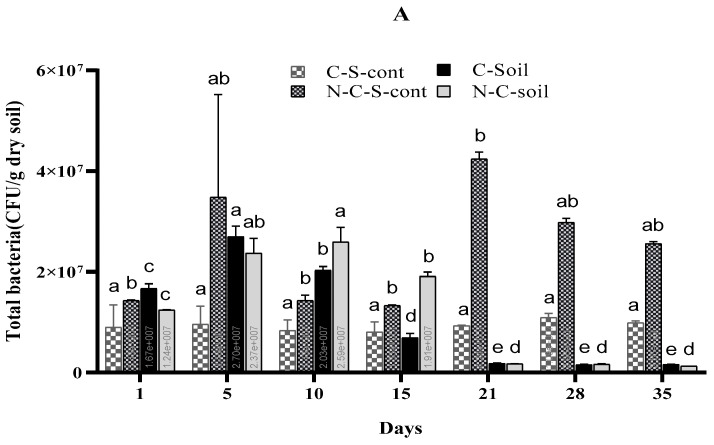

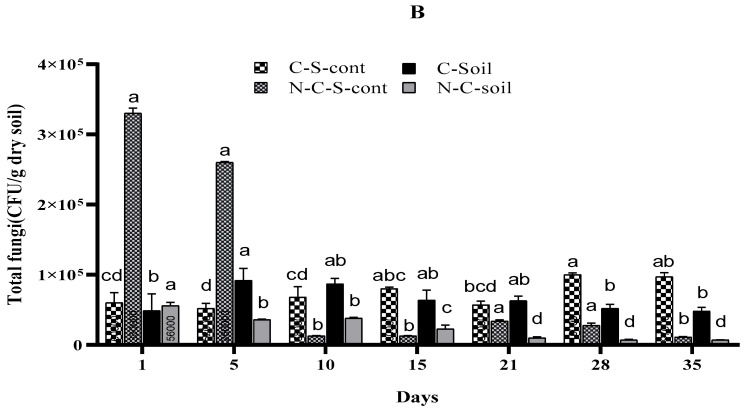
(**A**,**B**): The variation in a population of heterotrophic bacteria (**A**) and fungi (**B**) among different days of IMI exposure. Where: (CS) = cultivated soil, (NCS) = non-cultivated soil, (C-S-cont = cultivated soil control, and (N-C-S-cont) = non-cultivated soil control. Each point is the mean of three replications ± standard error. For a given sampling day, treatments sharing the same letter are not significantly different.

The population dynamics of heterotrophic bacteria exhibited fluctuations across different days of imidacloprid treatment (Figure 2A). In the control group of CS, the colony-forming units (CFU) remained relatively stable throughout the experimental duration, whereas in the NCS control, greater variability was observed, possibly due to the pre-treatment of the soil, involving drying and sieving. On day 5, the number of CFUs increased from the initial application for the IMI-treated soil but not for the CS control. After day 5, the CS soil showed a rapid decline in CFU until day 21, after which the CFU counts were uniform until the end of the study. The NCS treated with IMI had a similar profile, although CFU levels were relatively constant between days 5 and 10 and decreased afterward.

The fungal colony-forming units (CFU) varied significantly for the CS control, with low variability observed across the sample period, while a significant decrease in the NCS control was observed from a maximum recorded on day 1 (Figure 2B).

## 4. Discussion

### 4.1. Study Soil Characteristics

The long-term cultivated soil (CS) has significantly higher organic matter, total nitrogen, and available phosphorus compared to non-cultivated soil (NCS) due to crop residues and compost application. CS soil shows enhanced fertility indicators, reflecting active soil management and fertilization, while both soils have similar pH and EC, indicating stable conditions. Compared to typical agricultural soils in Europe, Central and Eastern Asia, and the Americas, these soils have lower organic matter, nitrogen, and phosphorus concentrations but are similar to soils in Saudi Arabia [35]. 

### 4.2. Imidacloprid (IMI) Concentration in Soil

The first-order kinetics, followed by the loss of IMI in both soil types, indicate an exponential decrease in concentration, with a half-life of 10.7 and 72.5 days in CS and NCS, respectively. The shorter half-life in CS suggests that cultivation practices may have influenced the loss rate of IMI from the soil. Factors such as microbial activity, soil composition, water flow rate, and agricultural practices that break up soil particles can all contribute to variations in dissipation rates [36].

The differences in IMI retention between CS and NCS imply that IMI is lost more rapidly in cultivated soils, likely due to percolation through tilled soil and higher content of organic matter. This indicates that a proportion of applied IMI in agricultural soils may enter the environment more rapidly after spraying compared to NCS. Understanding these kinetics and half-life values is crucial for assessing the environmental impact and persistence of IMI in different soil types, as well as understanding the efficacy of application.

The shorter half-life of IMI in CS compared to other NIs, such as clothianidin (CLO) and thiamethoxam (THIM), where the half-lives were estimated at 277–1386 and 7.1–92.3 days, respectively suggests that factors such as pesticide application techniques and overlaying plant species (maize and rice) might influence degradation rates [17]. Growing lettuce in CS may have contributed to the accelerated loss of IMI, as indicated in the previous studies of IMI [37].

### 4.3. IMI Uptake by Lettuce

The similar average concentration of IMI in lettuce roots for both soil types suggests that the plants reach an equilibrium concentration of IMI uptake irrespective of the initial external abundance, although the rate of achieving this equilibrium differs. The rapid increase in IMI concentration in the shoots within the first few days of exposure and the greater IMI concentration in shoots compared to roots and soil during the cultivation period indicate that IMI is more readily translocated into lettuce shoots.

These findings are consistent with studies on other NIs, such as rice plants grown in THIM-contaminated soils [38], and grapevines in IMI-contaminated soils [39]. However, the difference observed between the concentrations of CLO in maize roots and shoots, where no significant difference was found [17], suggests that the variations may be due to differences in plant species, the specific NI, or the application method of the plant protection product. Further studies are needed to explore these differences.

### 4.4. Root Concentration Factor (RCF) and Translocation Factor (TF)

The RCFs and TFs of IMI in this study are comparable to those of some pesticides, polycyclic aromatic hydrocarbons, perfluorinated alkyl acids, phthalate esters in different plants, and some pharmaceutical and personal care products in other studies. The results suggest that IMI exhibits a relatively strong translocation into the shoots of the lettuce plant, as indicated by its TF values in comparison to other compounds in various plants Table 3.

### 4.5. Effects of Imidacloprid on Soil Microbial Communities

The relatively stable CFU counts in the CS control group might be due to soil homogenization, which typically harbors fewer bacterial assemblages that are more resilient to soil disturbance. The increase in CFUs on day 5 for IMI-treated soil (except for the CS control) might suggest that IMI application stimulated CFU growth, possibly by decreasing some species, allowing for more diversity, or even acting as additional nutrients. However, the growth observed in the NCS control around the same time suggests that the microbial community in NCS might still be reaching equilibrium after soil processing.

The rapid decline in CFU counts in CS soil after day 5, and the subsequent stabilization until the end of the study indicates a possible adaptation or resilience in the microbial community. The similar profile observed in NCS soil, despite higher IMI levels, suggests that the CFU assemblage in NCS might have provided some resilience to IMI exposure. This observation is particularly interesting, as it contradicts the expectation of a more rapid decrease in CFUs with higher IMI exposure, indicating potential differences in microbial community responses between the two soil types.

Our findings align with those of previous studies, indicating that exposure to IMI can disrupt soil bacterial diversity in agricultural soils [45,46]. Negative impacts of IMI on total bacteria and soil biochemical parameters, particularly on nitrifying and N2-fixing bacteria, have been observed [46,47,48]. Conversely, some studies reported no significant negative effects of IMI on microbial activities [49], while others noted increases in bacterial populations alongside adverse effects on fungi and actinomycetes [50].

The mycorrhizal association between plants and fungi is a vital component of many plant species in lettuce, and arbuscular mycorrhizal fungi are known to aid in low-nutrient quality situations [51].

The impact on fungal communities varied, with IMI having a limited impact on the soil CFU for CS, while NCS IMI-treated soil showed a significant decrease in fungal CFU from the first day. The fungal communities in NCS are in a delicate balance with their environment, and the soil treatment’s impact on fungal CFU needs further investigation.

As both the treated and non-treated NCS soil show decreases in the fungal assemblage, linking any change to IMI exposure is not possible. However, the impact of the decrease in the fungal communities for NCS as a whole and what this may mean for the mycorrhizal association could be an interesting topic for future soil process studies.

Previous research observed an inhibitory effect of IMI on fungi in the soil after ~2 weeks of incubation [50,52]; however, other studies showed no negative effect of IMI on fungal numbers [49]. In our current study, both observations of IMI behaviour are correct depending on the soil type, although the clarity of fungal behaviour in NCS is less clear.

Consistent with our results, it has been demonstrated that IMI has a negative effect on urease, dehydrogenase, and phosphatase activities in soils [48]. There is a dose-dependent effect of IMI on urease activity, with increased activity at recommended doses in soil but decreased activity at higher concentrations [53]. Similarly, IMI application exhibited dose-dependent effects on phosphatase activity. However, unlike some activities, IMI had no inhibitory effect on dehydrogenase activity in sandy soil [25,50,52].

## 5. Conclusions

The results of this study have crucial implications, especially for understanding the dynamics of NI uptake and distribution in lettuce during agricultural practices. Understanding how these chemicals move within plants, such as lettuce, provides valuable insights that can be applied to agricultural practices, food safety assessments, and regulatory decision-making concerning the use of NIs. By shedding light on the uptake and movement of IMI within lettuce plants, this study contributes to a broader understanding of how NIs interact with vegetable crops. The information obtained can be used to assess the safety of food products, guide agricultural practices, and inform regulatory policies associated with NI use in farming. Knowledge of how long pesticides persist in the soil is crucial for assessing their long-standing effects on the environment, including on non-target organisms. Understanding non-target toxicity involves examining the unintended impacts of pesticides on organisms other than targeted pests. Soil microbes, being a critical component of ecosystems, are particularly sensitive to changes induced by pesticides. Soil microbes are essential for nutrient cycling in ecosystems, and their activities contribute to the decomposition of organic matter, nutrient release, and soil fertility, which affects soil health. This finding suggests that IMI application negatively affects soil microbes. This observation is in line with concerns regarding the potential ecological consequences of using certain pesticides.

There are also observations in the soil type and processing of the soil for experiments of uptake and plant systems, as the NCS types’ bacterial and fungal CFU counts showed variability during the period, which is something not observed with CSs. As fungal and bacterial interactions with plants are vital for the system’s health, experiments that use NCS may require a longer equilibrium time from sol processing to stabilize the soil before experimental activities to mitigate factors from the initial processing of the soil.

## Figures and Tables

**Table 1:** The basic characteristics of the two soils used in the current study.

Properties	NCS	CS
Organic matter (%)	0.55 b	1.79 a
Total nitrogen (%)	0.05 b	0.11 a
Available phosphorus (mg/kg)	0.11 b	0.69 a
Available potassium (mg/kg)	274 a	287 a
pH	7.34 a	7.56 a
EC (dS m^−1^)	0.70 a	0.69 a
Particle size distribution		
Sand (%)	51.1	39.1
Clay (%)	38.0	30.9
Silt (%)	10.9	30.0

**Table 2:** Root concentration factor (RCF) and translocation factor (TF) of IMI during 35 days of exposure. Each point is the mean of three replications ± standard error.

Days	Cultivated Soil (CS)		Non-Cultivated Soil (NCS)	
	RCF	TF	RCF	TF
1	0.3 ± 0.1	19.5 ± 0.3	0.3 ± 0.7	15.7 ± 14.9
5	0.5 ± 0.1	19.0 ± 0.2	0.5 ± 0.9	12.2 ± 19.4
10	1.0 ± 0.6	9.1 ± 0.2	0.5 ± 0.8	9.9 ± 13.0
15	1.4 ± 0.6	7.8 ± 0.2	0.5 ± 0.5	8.3 ± 6.9
21	3.2 ± 1.0	5.0 ± 0.3	0.5 ± 0.4	5.2 ± 2.4
28	2.5 ± 0.6	5.8 ± 0.3	0.6 ± 0.6	3.3 ± 1.1
35	2.5 ± 0.3	2.0 ± 0.2	0.5 ± 0.5	3.8 ± 0.9
Average	1.6	9.7	0.5	8.3

**Table 3:** RCFs and TFs of the same chemical compounds in different plants.

**Pesticides**	**RCFs**	**TFs**	**Plant Type**	**References**
parathion-methyl			Malabar spinach	[40]
propetamphos				
endosulfan				
fenthion				
difenoconazole	2		Rice and lettuce	[38,41]
perfluorinated alkyl acids				
imidacloprid		7.3		[38]
thiamethoxam		7.2		
chlorpyrifos	39	0.096 and 0.137	pakchoi and lettuce	[13]
acenaphthene	1.3		maize	[42]
fluorene	8.9			
phenanthrene	1.8			
perfluorinated alkyl acids			cabbage, zucchini, and tomato	
di-butyl phthalate		0.34	lettuce	[41]
di(2-ethylhexyl) phthalate		0.77		
mono-n-butyl phthalate		0.93		
mono (2 ethylhexyl) phthalate		0.57		
phenanthrene		0.006–0.12	Same plants	[43]
bisphenol A		0.051	collards	[44]
diclofenac		0.131		
naproxen		0.511		
nonylphenol		0.079		
parathion-methyl			Malabar spinach	[40]
propetamphos				
endosulfan				
fenthion				

## Data Availability

The data presented in this study are available upon request from the corresponding author.
